# Effect of Age on Variability in the Production of Text-Based Global Inferences

**DOI:** 10.1371/journal.pone.0036161

**Published:** 2012-05-08

**Authors:** Lynne J. Williams, Joseph P. Dunlop, Hervé Abdi

**Affiliations:** 1 Centre for Brain Fitness, Rotman Research Institute at Baycrest, Toronto, Ontario, Canada; 2 School of Behavioral and Brain Sciences, The University of Texas at Dallas, Richardson, Texas, United States of America; University of Leicester, United Kingdom

## Abstract

As we age, our differences in cognitive skills become more visible, an effect especially true for memory and problem solving skills (i.e., fluid intelligence). However, by contrast with fluid intelligence, few studies have examined variability in measures that rely on one’s world knowledge (i.e., crystallized intelligence). The current study investigated whether age increased the variability in text based global inference generation–a measure of crystallized intelligence. Global inference generation requires the integration of textual information and world knowledge and can be expressed as a gist or lesson. Variability in generating two global inferences for a single text was examined in young-old (62 to 69 years), middle-old (70 to 76 years) and old-old (77 to 94 years) adults. The older two groups showed greater variability, with the middle elderly group being most variable. These findings suggest that variability may be a characteristic of both fluid and crystallized intelligence in aging.

## Introduction

To understand spoken or written language, we need to integrate lexical, semantic, and contextual information and generate appropriate representations [1], [2]. Obviously, this process is highly dependent upon knowledge and memory [2]–[10], which are both sensitive to aging. But what happens to language comprehension when we age? The generally accepted view suggests that memory for textual details declines as memory declines with age [2], [4], [6], [11]–[13]. By contrast, however, older adults can access semantic information and understand complex linguistic representations as well as, or even better than, young adults in contexts where language comprehension is not dependent upon memory performance [2]–[10]. This apparent stability in older adults’ language comprehension performance is intriguing because text comprehension is a very complex activity (see e.g., [14]) that typically involves remembering the gist of the text rather than the surface details [15]–[21].

Kintsch [15] and colleagues suggested, that in order to remember the information in a text we need to reduce the amount of information by transforming the verbatim information into an abstract version of the text (see also [22], [23]). This abstract representation comes in the form of global inferences, which represent holistic concepts such as the theme or main point of a text [15], [16], [22]–[26]. These global inferences reduce the amount of information to be stored in memory because they integrate the text specific information with the individual’s world knowledge and experience (i.e., extra-textual information). Moreover, because global inferences represent generalized information (i.e., the text information is extended to contexts beyond the text itself, see e.g., [9]), we generate global inferences in order to fill informational gaps within the text and this allows us to incorporate the information from the text into our own world knowledge [27], [28].

Interestingly, the capacity to generate global inferences appears stable across age. For example, Ulatowska et al. [9] reported that there was no age difference in forming global representations of text in a longitudinal study of global inference generation in older adults. Similarly, Olness [29] found no differences between college-aged, middle-aged, and older adults in generating global inferences for didactic and non-didactic texts. Yet, there is growing evidence that knowledge structures thought to remain sable in aging–such as vocabulary and global inferences–may, in fact, be variable. For example, Christensen [30] found increased variability in older adults for measures of both memory, spatial, and reasoning skills (i.e., fluid intellectual abilities) as well as verbal abilities, including vocabulary (i.e., a crystallized intellectual ability). Similarly, Caskie, Schaie, and Willis [31] found considerable variability in verbal, spatial, and reasoning abilities in adults between 25 and 81 years of age. In addition, the patterns of variability were different for verbal abilities versus spatial and reasoning skills. In particular the changes in verbal abilities showed later onset, greater variability in the timing of onset, as well as greater variability in the overall rate of change. At the level of text comprehension, Hertzog, Dixon, and Hultsch [32] found significant variability in memory for textual information not accounted for by text-related factors in a group of seven elderly women. Likewise, Dixon and colleagues [33] reported an age increased variability in text recall for the stories used in the Logical Memory subtest of the Wechsler Memory Scale. Together all these findings suggest that an age-related increase in variability of the knowledge structures underlying linguistic ability and global inference generation may be a hallmark of cognitive aging, in the same way as the age-related increase in variability in reaction time, memory, and other cognitive abilities [34]–[42]. Therefore, we decided to investigate if age increased the variability of global inferences of participants. In order to do so, we measured age-related variability in generating global inferences among three groups of older adults.

## Results

Thirty-four participants between the ages of 62 and 94 years were divided into three age groups for the purpose of analysis. The young-old (Y-O) group consisted of 12 individuals (62 to 69 years of age), the middle-old (M-O) and old-old (O-O) elderly groups consisted of 11 participants each (70 to 76 and 77 to 94 years of age, respectively). Each participant gave 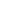 possible lessons for each of 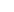 Aesop fables ([43], see Supplementary Information S.1). Each lesson was scored categorically according to the criteria outlined in the Method section 4.3.1. Data were analyzed using discriminant correspondence analysis (dica) [44]–[52].

Dica is a multivariate technique developed to classify observations described by qualitative and/or quantitative variables into a-priori defined groups and has been used to discriminate clinical populations, such as early versus middle stage Alzheimer’s disease [51] and autistic paranoia from paranoid schizophrenia [52]. Based on correspondence analysis (ca), dica is a type of principal component analysis (pca)–specifically tailored for the analysis of categorical data–that represents the rows and columns as points in (a high dimensional) space [45], [49]–[51], [53]–[57]. Just like pca, dica finds the most important dimensions of variance of the data. These dimensions are uncorrelated to each other and ordered by the amount of the data variance that they explain. Rows and columns can be plotted as maps by using their coordinates on these dimensions. In order to reveal the pattern of variables associated with group differences, dica analyzes a data table in which each row sums the behavior of the participants of a given group (see [51] for more information). Dica is then obtained from the ca of this summed table. This analysis reveals the similarities and differences in patterns of performance across the age groups. See Method section 4.3.3, File S2, Figure S1 and [44], [51], [58], [59] for more information.

The dica derived two factors accounting, respectively, for 85 percent and 15 percent of the data variance. The eigenvalues (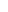), proportion of explained variance (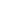), and the contributions of each variable and group to the total variance for Factors 1 and 2 are shown in Table 1. The higher the contribution, the more important that variable (or observation) is in defining a given factor.

**Table 1:** Eigenvalues (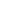), proportion of inertia (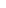), contributions of the age groups and scoring categories for Factors 1 and 2.

	Factor 1	Factor 2
Eigenvalue ( 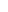 )^a^	0.0105	0.0018
Proportion of Explained variance ( 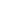 )	0.8539	0.1461
Contributions^b^		
Age Group		
Young Elderly	0.6449	0.0022
Middle Elderly	0.2118	0.4646
Old Elderly	0.1433	0.5332
Scoring Categories		
Switch Perspective		
Switch	0.1281	0.0004
Paraphrase	0.3344	0.0009
Linguistic Form^c^		
Proverb	0.0207	0.3624
Non-Proverb	0.0037	0.0653
Generalization Level (lesson 1)		
Extratextual	0.0648	0.0061
Text Specific	0.0570	0.0054
Generalization Level (lesson 2)		
Text Specific	0.0412	0.0017
Extratextual	0.0977	0.0041
Viewpoint Adopted (lesson 1)		
Main Character	0.0523	0.0102
Supporting Character	0.1081	0.0085
Other	0.0290	0.1517
Viewpoint Adopted (lesson 2)		
Main Character	0.0274	0.1221
Supporting Character	0.0347	0.2448
Other	0.0009	0.0162
Representation of Theme^d^ (lesson 1)		
Accurate	n/a	n/a
Inaccurate	n/a	n/a
Representation of Theme^d^ (lesson 2)		
Accurate	n/a	n/a
Inaccurate	n/a	n/a

a Note that in correspondence analysis, the eigenvalues (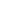) are never greater than 1.

b Contributions are the proportion of variance of a given factor explained by the age group or scoring category.

c Proverbial form for lesson 1 and lesson 2 combined due to similar profiles in previous versions of the analysis.

d Representation of Theme was included as a supplementary element, therefore, it did not contribute to the explained inertia of the factors.

### 2.1 Age-related Patterns of Global Inference Generation

**Figure 1:**
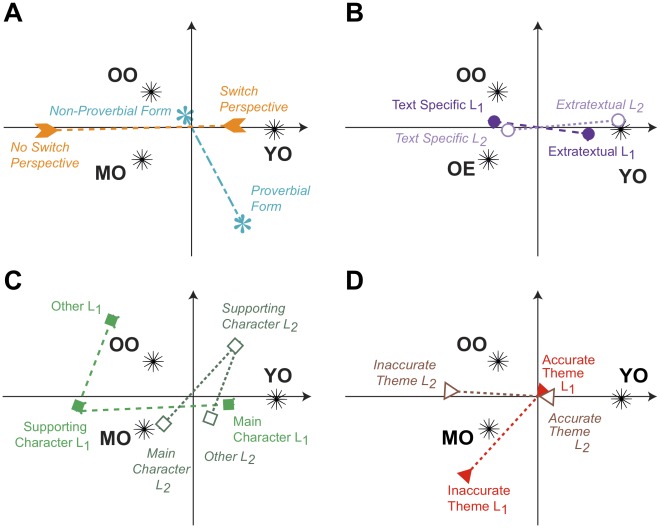
Discriminant correspondence analysis. Variables shown along Factors 1 and 2. Lambda (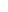) and tau (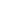) are the eigenvalues and the percentage of explained inertia (i.e., variance) for a given factor (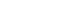, 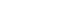; 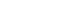, 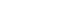). All sub-figures are plotted on the same scale along each factor. (A) Switch Perspective and Linguistic Form collapsed across both lesson types. Note that young elderly group switched perspectives between lesson types more frequently than the middle or old elderly groups. (B) Generalization Level for each lesson type. Note that the young elderly group produced extra-textual lessons more frequently. Extra-textual lessons incorporate information from outside of the text. (C) Character Viewpoint for each lesson type. Note that they young elderly group more frequently adopted the viewpoint of the main character for the best lesson (lesson 1) and the supporting character for the alternate lesson (lesson 2). (D) Representation of Theme was included as a supplementary element. Supplementary elements are variables that were not included in the calculations, but were projected into the space to see their placement along the factors. They are used to aid with interpretation. Note that the young elderly group more frequently produced lessons reflecting accurate fable themes for both lesson 1 and lesson 2. Note that in correspondence analysis, the eigenvalues are never greater than 1.

The dica uncovered age related patterns in lesson generation performance. Factor 1 separated the Y-O from the M-O and O-O groups (see Figure 1). Because dica reliably separated the Y-O from the other groups, the effect size is quite large and is detectable with our current sample size. However, to ensure that we could detect a reasonable effect size, we computed an a-posteriori effect size analysis using G*Power 3 [60], [61]. For the purpose of power analysis, multivariate discriminant analysis can be considered under the manova framework [62], [63]. With an 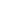 of.05 and achieved power (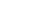) of.95, we had an effect size (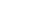) of.41. This effect size is equivalent to a critical Pillai’s 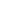 of 0.6 across the 3 groups, meaning that the between group-variance is 60% of the total variance. This effect size and critical 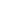 were considered adequate to be able to discriminate between the Y-O, M-O, and O-O groups.

The results of the dica are shown in Figure 1. The scoring categories are shown in separate displays to help reading the map. The variable contributing the most to Factor 1 is “switching perspectives between lesson types.” The young elderly group more frequently switched perspectives than the middle and old elderly groups. Success in switching between lesson 1 and lesson 2 is more strongly associated with a lesson 1 that incorporated information from outside of the text (i.e., extra-textual) and represented the main character’s viewpoint. Successful switches in perspective also were more frequently stated as proverbs and showed themes consistent with the fable for both lesson types. By contrast, the middle and older elderly groups switched perspectives less frequently than the young elderly group. Failure to switch perspective between lesson types was associated with maintaining the main character’s viewpoint for lesson 2 and producing text specific lessons for both lesson types (i.e., the information content of each lesson did not go beyond information stated explicitly in the fable). Switch failures also were characterized by more frequent use of non-proverbial linguistic forms (i.e., a concrete interpretation) and inaccurate representation of the fable theme for both lessons 1 and 2.

Factor 2 distinguished the middle and old elderly groups. The middle elderly group had a slightly greater tendency to maintain the main character’s perspective on lesson 2. Furthermore, the middle elderly group produced lesson 1 showing an inaccurate fable theme more frequently than those produced by the old elderly group. However, the old elderly group’s lesson 1 had a slightly greater tendency, on average, to adopt neither the main nor the supporting character’s perspectives. The old elderly group also tended to state both lessons in a non-proverbial form.

**Figure 2:**
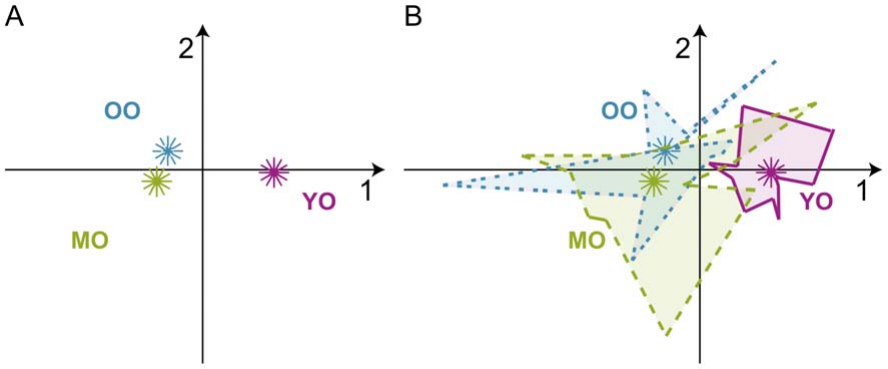
Discriminant correspondence analysis. Participants shown by age group along Factors 1 and 2. Lambda (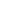) and tau (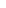) are the eigenvalues and the percentage of explained inertia (i.e., variance) for a given factor (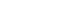, 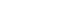; 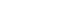, 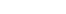). All sub-figures are plotted on the same scale along each factor. (A) Barycenters (weighted average) of the groups, (B) Convex hull. The convex hull represents the average performance of individual participants within each age group. Individual participants were projected into the dica space as supplementary elements. Supplementary elements are variables or observations that were not included in the calculations, but were projected into the space to see their placement along the factors. Note that in correspondence analysis, the eigenvalues are never greater than 1.

The performance of the groups and the individual participants by age group are shown in Figure 2. The young elderly participants clustered more tightly together, indicating that they were predominantly successful in switching perspectives. The tight grouping also suggests that the young elderly group showed less between participant variability in generating lessons. The middle and old elderly participants, by contrast, were more dispersed. Some of the middle and old elderly participants showed a pattern of lesson generation similar to the young elderly participants, while others did not. This suggests greater between participant variability, especially in the ability to switch perspectives between lessons 1 and 2.

### 2.2 Variability in Global Inference Generation

**Figure 3:**
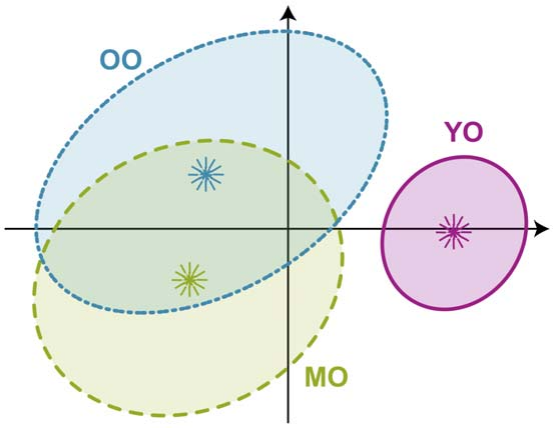
Discriminant correspondence analysis. 95% confidence intervals for age groups shown on factors 1 and 2. Lambda (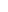) and tau (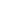) are the eigenvalues and the percentage of explained inertia (i.e., variance) for a given factor (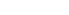, 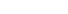; 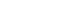, 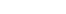). Confidence ellipses represent the variability within the group. Ellipses showing no overlap represent different populations. Note that in correspondence analysis, the eigenvalues are never greater than 1.

The variability in generating global inferences within the age groups was evaluated using a bootstrap procedure [64]–[66]. The bootstrap produced 95% confidence interval ellipses for each age group (see Figure 3; a description of the bootstrap is presented in the File S2.6.2). The area of a confidence interval ellipse represents the variability within each group. When the confidence ellipses do not overlap there is a significant difference between the groups at the 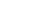 level. Consequently, the confidence ellipses show that the young elderly group is reliably different from the middle and old elderly groups because there is no overlap with the confidence ellipses of the other two groups. In addition, the size of the young elderly group’s ellipse is smaller, indicating that there is less variability within this group. Although the middle and old elderly groups were not reliably distinguished, the middle elderly group, surprisingly, had the ellipse with the greatest area indicating that the middle elderly group showed the most variability (see also Figures 2A and 2c for actual dispersion in group performance).

### 2.3 Quality of the DICA Model

We evaluated the quality of our dica model by computing the amount of variance explained by the dica (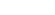, 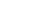; see Supplementary Information for details). We also evaluated how the model would generalize to new participants by using a jackknife procedure (also called “leave one out” procedure). The jackknife procedure [64], [67], [68] removes, in turn, each of the participants from the sample and performs a new dica on the remaining participants. The distance between the removed participant (projected into the new dica space as a supplementary element) and each of the groups is computed and the participant is assigned to the closest group (see [44] and [68] for more information about the jackknife in dica). The results of the jackknife are summarized in Table 2. The columns represent the original group assignment and the rows represent the dica assignment. From Table 2, we found that of the 34 possible assignments, only 13 were correct. The young elderly participants were more reliably assigned to their group (9 out of 12 correctly assigned) than participants from the middle and old elderly groups (2 correct assignments out of 11 participants for each group). This difference in classification reflects the larger variability of the middle and old elderly groups.

**Table 2:** Actual versus dica participant classification into young elderly, middle elderly, and old elderly groups.

dica Assigned Group	Actual Group		
	Young Elderly	Middle Elderly	Old Elderly
Young Elderly	*9*	3	4
Middle Elderly	1	*2*	5
Old Elderly	2	6	*2*

Note: Numbers in italicized print represent correctly classified participants.

## Discussion

Although most studies examining cognitive performance variability in the elderly have examined skills that are known to decrease with age (e.g., fluid intelligence abilities, reaction time (rt) or memory [39], [40], [69]–[83]), skills that remain stable or improve across age (i.e., crystallized intelligence) also show inter-trial variability. However, this age-associated pattern of variability may differ between the two intelligence domains. For example, variability in rt for speeded tasks shows that older adults are consistently more variable than younger adults [81]–[83] and that this increased rt variability is associated with poorer cognitive performance in normally aging older adults [84]–[86]. The general increase in variability in the M-O and O-O groups relative to the Y-O group supports this view and may be associated with the older two groups’ general difficulty in switching perspectives between lesson 1 and lesson 2.

By contrast, when older adults show increased variability in gist recall accuracy (rather than rt or detail recall), this increase in variability tends to be associated with poor health, rather than normal age-related change [32], [87]. In normally aging adults, increased item-to-item variability in non-speeded tasks (such as gist recall) is associated with higher mean performance and may actually be an indicator of learning rather than decline [76], [88]. The finding that the M-O group showed greater variability than the O-O group suggests that, at least in non-speeded tasks, increased variability may not be completely maladaptive. The strict view of increased variability indicating cognitive decline predicts a linear association between variability and age (see e.g., [81]–[86]), yet the current data do not show this pattern. Rather, they suggest the possibility varying patterns of variability at different life stages, especially given that the Y-O, M-O, and O-O individuals were cognitively normal and successfully performed the task.

If we consider that learning may also be a mechanism for increased variability in aging, then the M-O group would be expected to show the greatest amount of variability because this group has the largest proportion of recently retired individuals undergoing a major life change. For example, Adam and colleagues [89], [90] have found sudden decreases in cognitive functioning immediately following retirement, a pattern which suggests that there may be an increase in variability in cognitive performance around this time. Such a change in variability would be similar to the recursive increases in variability and subsequent plateauing during periods of social and cognitive development during childhood and adolescence [88].

Although showing increased variability associated with age, the current results show a mixed pattern. This suggests that multiple mechanisms may underlie the increase in performance variability for crystallised intellectual abilities in older adults and that the relationship between age and variability may not be as straightforward as with fluid intellectual skills. Nevertheless, these findings show that variability with age may not be just an indicator of decline, but may also signal new learning. As Garrett et al. [91] so aptly said, “variability is more than just noise” (p. 4914).

## Methods

### 4.1 Participants

Thirty-four participants between the ages of 62 and 94 years were divided into three age groups for the purpose of analysis. The young elderly (Y-O) group consisted of 12 individuals (62 to 69 years of age), the middle (M-O) and older (O-O) elderly groups consisted of 11 participants each (70 to 76 and 77 to 94 years of age, respectively). All participants were highly educated, with an average of 15 years of formal education. All participants were living in the community and were self-reported native English speakers. None exhibited clinical signs of impaired cognitive performance as tested by the 7 Minute Screen [92], [93]. All participants scored within normal age limits on a hearing screening that included the Erber Sentences [94], CID Sentences [95], and a self-report of hearing loss. All subjects made no errors on a visual narrative screener where they read aloud an additional fable typeset in the same font as the stimulus fables. This study was approved by the Internal Review Board (IRB) of the University of Texas at Dallas. All participants gave written informed consent. Table 3 gives the participant characteristics.

**Table 3:** Participant characteristics.

	Age (yrs)		Education (yrs)		Similarities^a^		LM I^b^	
	Mean	SD	Mean	SD	Mean	SD	Mean	SD
Young Elderly	65.58	(2.27)	16.25	(3.11)	27.50	(4.19)	18.67	(2.46)
Middle Elderly	72.18	(2.32)	16.91	(3.39)	27.27	(2.97)	18.45	(2.98)
Old Elderly	82.73	(5.41)	14.36	(3.26)	22.40	(8.46)	18.10	(2.64)
	LM II^b^		Trails (B 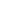 A)^c^		WCST (Total Correct)^d^			
	Mean	SD	Mean	SD	Mean	SD		
Young Elderly	26.00	(7.31)	29.13	(22.48)	50.58	(7.56)		
Middle Elderly	23.00	(8.98)	43.06	(35.06)	43.36	(8.37)		
Old Elderly	14.80	(8.98)	125.92	(98.87)	42.40	(12.21)		

Note: All scores from psychometric testing are represented as raw scores for the given subtest;

a Wechsler Adult Intelligence Scale III [110];

b Wechsler Memory Scale III [111];

c Trail Making Test [112];

d Wisconsin Card Sorting Test, 64 Card Version [113].

### 4.2 Stimuli and Task

We selected twelve short narratives from George Townsend’s translations of Aesop’s fables [43]. We used fables because cultural knowledge is transmitted via their didactic form. This transmission of cultural knowledge takes the form of a lesson or moral (i.e., types of global inferences). In addition, the role of the fables in transmitting knowledge or “general truths” gives to the fables a similar function to proverbs in discourse. However, unlike proverbs, fables require the theme, lesson, or moral to be inferred from the characters’ actions and their consequences. Meaning in proverbs, by contrast, is derived from the text itself and not from its application to real-world contexts because proverbs are already stated in a global inference-like format [9], [96], [97]. Because fables are didactic, readers can interpret them at two levels: literally, at the level of the text itself (i.e., a textual interpretation), or metaphorically, as a guide to culturally appropriate behavior in real-life contexts (i.e., an extratextual interpretation; [98]–[102]). Given that multiple interpretations of each fable is possible, fables can be, at least in part, interpreted as each reader chooses [103] and therefore interpretations of a given fable can vary with the reader, the information that is chosen as salient during comprehension (e.g., a given character’s actions), and the overall level of generalization (i.e., textual versus extratextual).

All fables employed two characters, contained three episodes (i.e., setting, action, and resolution components), were between 10 and 21 propositions in length [15], and contained no mixture of anthropomorphized animal and human characters. We then modified the fables to exclude specific mention of character attributes (e.g., lazy, wise, etc.) and any specific mention of the moral or lesson. Fables are shown in File S1. We asked participants to generate two different lessons or morals for each of the 12 fables. We instructed participants to first give what they considered to be the “best” lesson for the fable (lesson 1). We then asked participants to generate a second possible lesson for each fable that reflected a different interpretation or perspective (lesson 2). The examiner read the fable to participants and a card with the printed fable was within view during generation of both lessons to minimize memory demands.

### 4.3 Analysis

#### 4.3.1 Response coding

Lessons were scored categorically according to: (a) whether there was a switch in perspective between lesson 1 and lesson 2, (b) whether the lesson reflected text specific or extratextual content [9], [29], (c) whether the lesson portrayed the viewpoint of the main or of the supporting character [98], and (d) whether the lesson was given in the form of a statement or proverb, that is, a literal or metaphorical interpretation [104]. The accuracy or semantic fit of each lesson theme was scored in reference to the original fable. Representation of theme was not included as an active variable in the analysis due to the high degree of accurate semantic representation produced by all three age groups (91% accurate). Table 4 shows further definitions of the scoring categories with examples.

**Table 4:** Scoring Criteria for lesson 1 and lesson 2 Lesson Responses.

	Definition	Example
Switching Perspective		
Switch	Lesson 2 represents a different idea, theme, or viewpoint than lesson 1	lesson 1: Be careful whom you trust; lesson 2: Sometimes good intentions go astray
Fox & Goat [Px O-O 7]		
Paraphrase	lesson 2 represents the same general idea, theme, or viewpoint as lesson 1; Lessons may be stated in a different linguistic form.	lesson 1: United we stand, divided we fall; lesson 2: Strength in numbers
Father & Sticks [Px M-O 8]		
Generalization Level^a^		
Text Specific	Lesson remains tied to the characters, actions, or events of the fable.	Make sure that what you say is true, because you may need to prove it
Boasting Traveller [Px Y-O 7]		
Extratextual	Lesson extends beyond the actions and events of the fable. Contains inferred information not contained in the original fable.	Gold and rubies are not the only treasures in this world
Farmer & Sons [Px Y-O 9]		
Viewpoint Adopted		
Main Character	Lesson is stated from the viewpoint of the main fable character	When you cry, make sure its true. Or when you do anything or want help from anyone, make sure its true
Shepherd Boy [Px Y-O 3]		
Supporting		
Character	Lesson is stated from the viewpoint of the supporting fable character	People get tired of being made fools of and they eventually learn not to respond
Shepherd Boy [Px Y-O 10]		
Other	Lesson is stated from a perspective that does not distinctly adopt the viewpoint of either the main or supporting fable characters; Lesson may reflect a mixed or indeterminate viewpoint, or is not character specific	The moral of the story is that each of us has a gift
		Crane & Peacock [Px M-O 7]
		That there’s not, um, a same fix for each person
		Father & Sticks [Px Y-O 5]
Linguistic Form		
Proverbial	Lesson is stated in the form of a proverb	United we stand, divided we fall
Father & Sticks [Px O-O 2]		
Non-Proverbial	Lesson is not proverbial	Try not to take on more than you can handle in detail. Try and break it down in smaller amounts and complete each effort singly
Father & Sticks [Px O-O 7]		

Representation of Theme^b^		
Accurate	The theme represented in the lesson is appropriate for the fable	The proof of the pudding is in the eating. If you have to if you have to boast, then you should be able to perform what you’re saying
Boasting Traveller [Px O-O 2]		
Inaccurate	The theme represented in the lesson is inappropriate for the fable	If you pick at a sore, it will get worse
Miser [Px M-O 11]		

Y-O = Young elderly, M-O = Middle elderly, O-O = Old elderly;

a Modified from Olness [29];

b Scoring category included as supplementary variable in the dica analysis due to high frequency of accurate responses. A supplementary variable is one that was not included in the analysis, but was placed in the display to aid with interpretation of the factors.

#### 4.3.2 Inter-rater reliability

Inter-rater reliability was analyzed on a random 20% of the data by comparing the first author’s coding with the code ratings of a second trained rater. Point-by-point agreement was 79%. A Cohen’s Kappa was calculated to correct for chance agreement (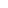 = 0.621), corresponding to a “substantial” rating of agreement [105].

#### 4.3.3 Statistical analysis

We used discriminant correspondence analysis (dica) to analyze the coded lesson responses. Dica combines the features of correspondence analysis (ca) and discriminant analysis ([44], [106]; see also [51] for a tutorial on language oriented applications). Correspondence analysis (ca) is a type of principal component analysis (pca)–specifically tailored for the analysis of categorical data–that represents the rows and columns as points in a (high dimensional) space [45], [49], [50], [53]–[55]. In addition, ca (and consequently, dica) can handle data sets with few observations described by many nominal variables [44], [45], [51], [107].

Just like pca, ca finds orthogonal factors or dimensions that reveal the patterns and the associations between the row and column profiles. The importance of the factors is determined by their inertia (i.e., a quality akin to variance), denoted by 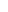 and the proportion of explained inertia, denoted by 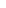. ca converts contingency tables into visual displays (i.e., maps) in which the row profiles and column profiles represent points in the display. The proximity of the points within the display represents their degree of associa’tion. Points distributed more closely in space are more strongly associated than those that are farther apart. In addition, ca places no constraints on the data; therefore, the pattern seen in the maps represents associations contained within the data and not those superimposed by an external model [47], [48].

Dica is a multivariate technique developed to classify observations described by qualitative and/or quantitative variables into a-priori defined groups and therefore adds a discriminative component to ca. Here, we used dica to analyze lesson 1 and lesson 2 responses and to classify participants into pre-defined age categories: young-old (Y-O), middle-old (M-O) and old-old (O-O) groups.

For the dica, participants were grouped into the three age groups. Then, the pattern of performance of the participants in each group was combined into its common pattern of performance (see [51] for more information on how the common pattern is developed). Table 5 shows the age-group by lesson response contingency table, the common pattern of performance used for the dica in the current study.

**Table 5:** Frequency of occurrence of scoring categories by lesson type for the young elderly, middle elderly, and old elderly groups (contingency table input into dica).

	Switch Perspective		Linguistic Form	
	Switch	Paraphrase	Proverbial	Non-Proverbial
Young Elderly	119	25	25	119
Middle Elderly	87	45	22.5	109.5
Old Elderly	89	43	14.75	117.25

	Generalization Level			
	lesson 1		lesson 2	
	Extratextual	Text Specific	Extratextual	Text Specific
Young Elderly	76	68	51	93
Middle Elderly	58	74	34	98
Old Elderly	57	75	36	96

	Character Viewpoint			
	lesson 1			lesson 2
	Main	Supporting	Other	Main
Young Elderly	113	19	12	72
Middle Elderly	91	28	13	78.67
Old Elderly	89	26	17	69.34

	Character Viewpoint			
	lesson 2			
	Supporting	Other		
1−3 Young Elderly	57	15		
Middle Elderly	39.66	13.67		
Old Elderly	50.32	12.34		

We then ran a ca on the common performances, which allowed us to examine the similarities and differences in patterns of performance across the age groups. ca and dica also can be used to estimate the amount of variability within and between each category. To do this 95% confidence ellipses are constructed using a bootstrap resampling technique ([108], [109]; see also File S2.6.2). A detailed mathematical appendix is included in the Supplementary Information.

## Supporting Information

Figure S1 (TIFF)

File S1 Supporting Information PDF file.
(PDF)

File S2 (PDF)
