# Supplementary figures and images for: Effect of Age on Variability in the Production of Text-Based Global Inferences
Source: PLoS One. 2012 May 8;7(5):e36161. doi: 10.1371/journal.pone.0036161 (PMC3348169; doi:10.1371/journal.pone.0036161)

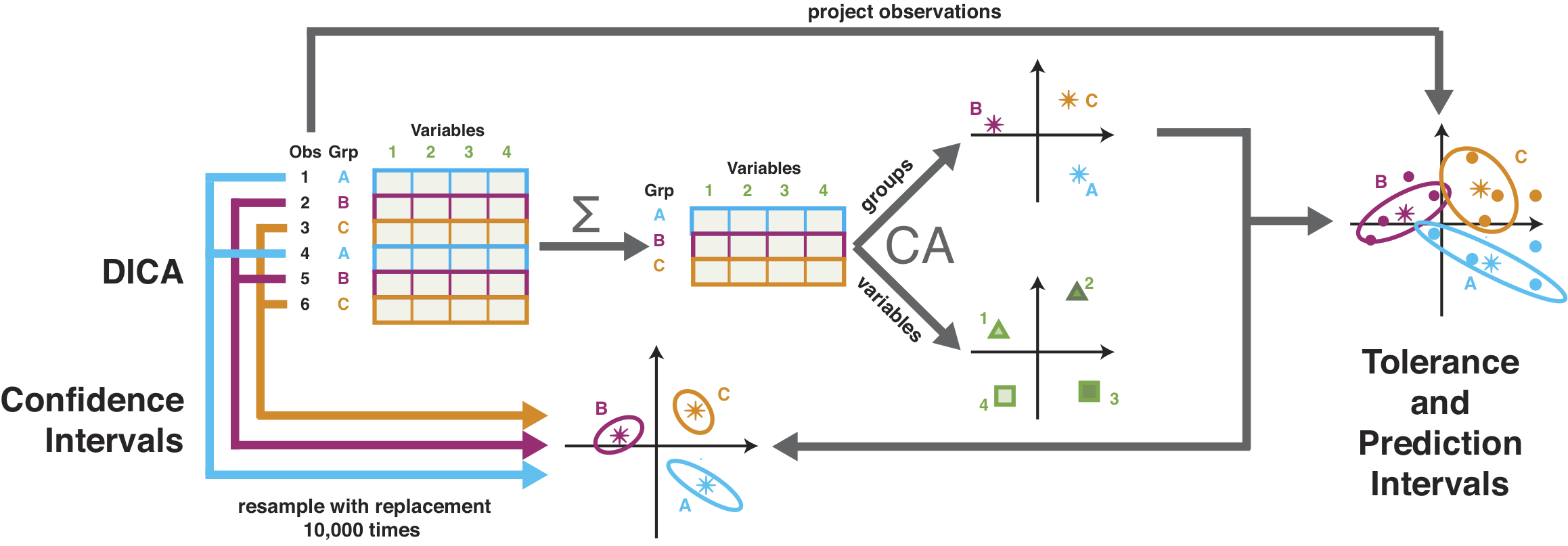

Supplement: Figure S1 — (TIFF) [file pone.0036161.s001.tiff]
