# Supplementary material for: Effect of Age on Variability in the Production of Text-Based Global Inferences
Source: PLoS One. 2012 May 8;7(5):e36161. doi: 10.1371/journal.pone.0036161 (PMC3348169; doi:10.1371/journal.pone.0036161)
Supplement: File S1 — Supporting Information PDF file. (PDF) [file pone.0036161.s002.pdf]

Supplementary Information
Effect of Age on the Variability in the Production of Text-Based Global Inferences

Lynne J. Williams[1,*], Joseph P. Dunlop[2], Hervé Abdi[3,*]

1 Lynne J. Williams Centre for Brain Fitness, Rotman Research Institute, Baycrest, Toronto, ON, CANADA lwilliams@research.baycrest.org

2 Joseph P. Dunlop School of Behavioral and Brain Sciences, The University of Texas at Dallas, MS: GR4.1, 800 West Campbell Road, Richardson, TX USA joseph.dunlop@utdallas.edu

3 Hervé Abdi School of Behavioral and Brain Sciences The University of Texas at Dallas MS: GR4.1 800 West Campbell Road Richardson, TX 75080-3021 USA herve@utdallas.edu

∗ **E-mail: lwilliams@research.baycrest.org, herve@utdallas.edu**

Fables

S1.1 Boasting Traveller

Once upon a time, a world traveller boasted of performing superhuman feats in the places he had visited. The more he boasted, the more people gathered around to hear his stories. Once a small group of listeners had gathered, he told them, "While in Caracas, in front of a group of people like you, I leaped farther than any man in history. I bet no one can match my jump." Doubtful of the traveller's claims, one of the bystanders interrupted him, saying,"If your feat is true, you don't need any witnesses, just leap for us."

S1.2 Crane and Peacock

Once upon a time, a peacock was admiring his gorgeous tail. Preening, he marvelled at how the colors flashed in the sunlight. Seeing a crane, he made fun of the drabness of the crane's coloring. "I am colored like the robes of kings in gold and purple and all the colors of the rainbow. You do not have any color, so

you are no where near as beautiful or good as I am." "True, I may not be beautiful," replied the crane, "but I can fly among the heavens, while you are limited to the ground."

S1.3 Farmer and Sons

A farmer found out that he was dying. He wanted his children to treat his farm with the same care that he did. He loved his farm and wanted it to thrive. He called his sons to his bedside and said, "My children, you must know that there is a treasure hidden in my vineyards." After the man died, the children dug the ground trying to find this great wealth. They found no treasure, but amazingly enough, the vineyards produced a wonderful amount of fruit.

S1.4 Father and Sticks

A father once had sons who always argued with each other. Frustrated with their fighting, he pleaded with the boys to stop. When his pleading did not work, he was determined to show his sons the value of his advice. One day, the father asked his sons to bring him a bundle of sticks. Once they brought the bundle to him, the father asked his sons to break it. One by one, each son tried to break it, but even using all their strength, no one could. After the sons had failed, the father opened the bundle of sticks and gave one stick to each boy. With only one each, the sons had no trouble breaking the sticks.

S1.5 Fox and Goat

Once upon a time, a fox was walking through a field and slipped into a well. Frightened, the fox tried to climb out but the sides of the well were too steep. Later that day, a goat wandered by looking for some water to drink. Finding the fox, the goat asked if he knew where there was some water. With a sly thought, the fox told the goat of the magical properties of the well's water. The fox told the goat to jump into the well in order to get his drink. Believing the fox, the goat jumped in. As the goat was enjoying his drink, the fox told of his difficulty getting out of the well. Panicked, the goat cried in frustration at being stuck in the well. The fox said, "Do not worry, I have a plan. If you lean against the wall, I will run up your back to escape. And don't worry, I will help you get out after me." Reassured, the goat placed his front hooves against the wall. The fox quickly ran up his back, however, instead of helping the goat he kept on running.

S1.6 Lion and Mouse

Once upon a time, a mouse ran across a sleeping lion's face. Startled, the lion woke up and in his anger intended to kill the mouse. The mouse begged the lion not to harm him and said, "If you do not hurt me, I am sure I can find a way to return your favor." The lion laughed because he did not believe the mouse could ever help him, but he was intrigued and let the mouse go. Sometime later, a group of hunters caught the lion and tied him up with ropes. The lion roared and roared, but no one responded to his cries. When the mouse finally heard the lion's roar, he came to see what was the trouble. Seeing the lion tied up, the mouse chewed through the ropes.

S1.7 The Miser

Once upon a time, a man sold everything that he had and bought a lump of gold. Not wanting to lose his treasure, he buried the gold beside an old wall. He visited the spot every day to make sure that his gold was safe. One day, one of his workmen noticed the man's many visits to the same spot. Curious, the workman went to see what his employer was looking at. Discovering the man's secret, the workman dug up his employer's treasure. Not long after, the man discovered that his gold was gone.

S1.8 Old Woman and Physician

Once upon a time, an old woman became blind and she asked a doctor to restore her vision. They agreed that the doctor would only be paid if the woman regained her sight. Every day, the doctor came and applied an ointment to her eyes. On every visit, the doctor took some of the woman's valuables with him. After many treatments with the ointment, the doctor gave the woman a different medicine that restored her sight. When the woman opened her eyes, she could not see any of her valuable belongings, so she refused to pay. The doctor took the woman to court where she told the judge, "I did promise to pay the doctor when my sight was restored. He claims that I am healed, but I say I am still blind. When I lost my sight, I could see my valuables. Now, although he swears he has cured me, I am not able to see a single thing."

S1.9 Shepherd Boy

Once upon a time there was a young shepherd who was watching a flock of sheep near a village. Being young, he got bored easily so, several times the boy cried, "Wolf! Wolf!" The villagers came running to his aid. When they arrived, the boy would laugh because there was no real wolf and he called them just to pass the time. One day, a wolf attacked. The shepherd boy cried, "Wolf! Wolf! A wolf is eating my sheep!" but none of the villagers came to help him.

S1.10 Sick Lion

Once upon a time, an old lion discovered he could not catch his food by hunting anymore. In order to not go hungry, he decided to get his food by trickery. He pretended that he was very sick. The other animals, feeling terrible for the old lion, went to his cave in order to help him in his recovery. The old lion, hungry, quickly ate his visitors and waited for the next one to come so that he could have another meal. The fox, noticing that many animals were missing, went to visit the lion. She saw all the footprints going into the cave, but saw none coming out. Suspicious, the fox called out to the lion and asked how he was doing. "I am not well," responded the lion, "please come in so I can talk to you." "No, thank-you," said the fox, "I see many footprints going into your cave, but I do not see any coming out."

S1.11 Turtle and Rabbit

Once upon a time, a rabbit made fun of a turtle's short legs and slow pace. In good humor, the turtle teased, "These short legs will beat you in a race any day." Thinking that he was unbeatable, the rabbit accepted the challenge. Together the rabbit and the turtle decided that the fox should design the course. Once the course was ready, the rabbit and the turtle started the race at the same time. The rabbit put on a quick burst of speed, leaving the turtle behind. Not seeing the turtle behind him, the rabbit decided to take a nap. Meanwhile, the turtle pushed ahead with his persistent slow pace. After some time, the rabbit woke up and realized how late it was, so he nervously decided to use his full speed in order to win the race. Just as he was about to cross the finish line, he noticed that the turtle had already crossed the line and was ready to greet the rabbit.

S1.12 Two Roosters

Once upon a time there were two roosters who always fought over the command of the roost. One day, they had a really big fight and one of the roosters finally beat the other into submission. The rooster who lost hid in a quiet corner of the roost. The winner went to the top of a fence and started crowing and flapping his wings in order to boast of his victory. All of a sudden, an eagle swooped down and snatched away the winner. Later, the other rooster came out from his corner. Now, being the only rooster, he ruled the roost.

DICA: Formal Presentation

The goal of discriminant correspondence analysis (DICA) is to predict group membership of observations which are described by nominal variables (or by variables which represent the *amount* of some quantity). DICA is shown schematically in Figure S1.

S1.13 Notations

We have I observations each described by J variables. The values of the variables for the observations are stored in an I by J data matrix denoted $\mathbf{X}$. The observations of $\mathbf{X}$ are partitioned into N *a-priori* groups of interest with I_n being the number of observations of the nth group (and so $\sum_n^N I_n = I$). The elements of $\mathbf{X}$ are assumed to be positive or zeros and we assume that there are no empty rows or columns (i.e., rows or columns with only zero values).

S1.13.1 Notations for the Groups (Rows)

We denote by $\mathbf{Y}$ the I by N design matrix for the groups describing the rows of $\mathbf{X}$: $y_{i,n} = 1$ if row i belongs to group n, $y_{i,n} = 0$ otherwise.

S1.14 Step 1: Compute the group × variable matrix

The first step of DICA is to compute the N by J matrix of the total of each group. This matrix is called $\mathbf{S}$ and it is computed as

$$\mathbf{S} = \mathbf{Y}^{\mathsf{T}}\mathbf{X} \ . \tag{1}$$

The grand total of $\mathbf{S}$ is denoted s_{++} (i.e., $s_{++} = \mathbf{1}^{\mathsf{T}}\mathbf{S}\mathbf{1}$). From matrix $\mathbf{S}$ we compute a matrix of barycentric row *profiles* denoted $\mathbf{R}^*$ and computed as

$$\mathbf{R}^* = \mathsf{diag}\{\mathbf{S}\mathbf{1}\}^{-1}\,\mathbf{S} \tag{2}$$

where the diag operator transforms a vector into a diagonal matrix when applied to a vector and extract the vector of the diagonal elements when applied to a matrix. A row of $\mathbf{R}^*$ is a profile because it is made of non-negative numbers whose sum is equal to one. When transformed into profiles, two rows can be compared independently of their overall level. The *masses* of the barycenters are proportional to the sum of the corresponding groups. Specifically, the N by 1 group mass vector denoted $\mathbf{b}$ is computed as

$$\mathbf{b} = \mathbf{S}\mathbf{1} \times s_{++}^{-1} \tag{3}$$

The diagonal barycenter mass matrix is obtained from the barycenter mass vector as

$$\mathbf{B} = \mathsf{diag}\{\mathbf{b}\}\ . \tag{4}$$

The "grand barycenter," denoted $\mathbf{c}$, is the overall barycenter of matrix $\mathbf{R}^*$, it is computed as

$$\mathbf{c} = \mathbf{S}^{\mathsf{T}}\mathbf{1} \times s_{++}^{-1}\ . \tag{5}$$

The *weights* of the columns are inversely proportional to their frequency. The weights are stored in a J by 1 vector denoted $\mathbf{w}$ and the corresponding J by J diagonal matrix is denoted $\mathbf{W}$. Specifically, $\mathbf{W}$ and $\mathbf{w}$ are computed as:

$$\mathbf{W} = \mathsf{diag}\{\mathbf{c}\}^{-1} \quad \text{and} \quad \mathbf{w} = \mathsf{diag}\{\mathbf{W}\}\ . \tag{6}$$

S1.15 Step 2: Correspondence Analysis of the $\mathbf{R}^*$ Barycenter Matrix

The $\mathbf{R}^*$ matrix is then analyzed using CA. Specifically, the first step of the analysis is to *center* $\mathbf{R}^*$ in order to create a matrix of centered profiles. This matrix, denoted $\mathbf{R}$, is computed as

$$\mathbf{R} = \mathbf{R}^* - \mathbf{1}\mathbf{c}\ , \tag{7}$$

(with $\mathbf{1}$ being a N by 1 vector of 1s). Then, the matrix $\mathbf{R}$ is analyzed with a generalized singular value decomposition under the constraints provided by the matrices $\mathbf{B}$ (masses for the N groups) and $\mathbf{W}$ (weights for the columns) as [1–4]:

$$\mathbf{R} = \mathbf{P}\boldsymbol{\Delta}\mathbf{Q}^{\mathsf{T}} \quad \text{with} \quad \mathbf{P}^{\mathsf{T}}\mathbf{B}\mathbf{P} = \mathbf{Q}^{\mathsf{T}}\mathbf{W}\mathbf{Q} = \mathbf{I} \ , \tag{8}$$

where $\boldsymbol{\Delta}$ is the L by L diagonal matrix of the singular values (with L being the number of non-zero singular values), and $\mathbf{P}$ (respectively $\mathbf{Q}$) being the N by L (respectively J by L) matrix of the left (respectively right) generalized singular vectors of $\mathbf{R}$ (the singular vectors are also called *eigenvectors* and the the squared singular values are also called *eigenvalues*; see for details, [5]).

S1.15.1 Row Factor Scores

The N by L matrix of factor scores for the groups is obtained as

$$\mathbf{F} = \mathbf{P}\boldsymbol{\Delta} = \mathbf{R}\mathbf{W}\mathbf{Q} \ . \tag{9}$$

The variance of the columns of $\mathbf{F}$ is given by the square of the corresponding singular values (i.e., the "eigen-value" denoted λ, these are stored in the diagonal matrix $\boldsymbol{\Lambda}$). This can be shown by combining Equations 8 and 9 to give:

$$\mathbf{F}^{\mathsf{T}}\mathbf{B}\mathbf{F} = \boldsymbol{\Delta}\mathbf{P}^{\mathsf{T}}\mathbf{B}\mathbf{P}\boldsymbol{\Delta} = \boldsymbol{\Delta}^2 = \boldsymbol{\Lambda} \ . \tag{10}$$

S1.15.2 Column Factor Scores (Loadings)

In correspondence analysis, the roles of the row and the columns are symmetrical: They can be represented in the same map because they have the same variance. Therefore, the columns are described by *factor scores* which can also be interpreted as loadings. Column factor scores are used to identify the variables important for the separation between the groups. In DICA, the column factor scores are computed as (*cf.* Equations 8 and and 10):

$$\mathbf{G} = \mathbf{W}\mathbf{Q}\boldsymbol{\Delta} \ . \tag{11}$$

S1.16 Projection of the Observations in the Discriminant Space

The I rows of matrix $\mathbf{X}$ can be projected (as "supplementary" or "illustrative" elements) onto the space defined by the factor scores of the barycenters. The first step is to transform $\mathbf{X}$ into a matrix of centered row profiles called $\mathbf{L}$ and computed as:

$$\mathbf{L} = \left(\mathsf{diag}\{\mathbf{X1}\}^{-1}\mathbf{X}\right) - \mathbf{1c}, \tag{12}$$

(with $\mathbf{1}$ being an I by 1 vector of 1s). Then from Equations 8 and 9, we find that matrix $\mathbf{WQ}$ is a projection matrix. Therefore, the I by L matrix $\mathbf{H}$ of the factor scores for the rows of $\mathbf{X}$ can be computed as

$$\mathbf{H} = \mathbf{LWQ} = \mathbf{LG}\boldsymbol{\Delta}^{-1}. \tag{13}$$

These projections are barycentric, because the weighted average of the factor scores of the rows of a group gives the factor scores of the group. Specifically, if we define $\mathbf{M}$ as the mass matrix for the observations as

$$\mathbf{M} = \mathsf{diag}\{\mathbf{m}\} = \mathsf{diag}\left\{\mathbf{X1} \times s_{++}^{-1}\right\} \ . \tag{14}$$

Note that the factor scores of the barycenters are the barycenter of the factor scores of the projections of the observations. This is shown by first computing the barycenters of the row factor scores as (*cf.* Equation 2) as

$$\overline{\mathbf{H}} = \mathsf{diag}\{\mathbf{YM1}\}^{-1}\mathbf{YMH} \ , \tag{15}$$

then plugging in Equation 13 and developing. Taking this into account, Equation 9 gives

$$\overline{\mathbf{H}} = \mathsf{diag}\{\mathbf{YM1}\}^{-1}\mathbf{YMXWQ} = \mathbf{RWQ} = \mathbf{F} \ . \tag{16}$$

S1.17 Quality of the Prediction

The performance, or quality, of the prediction of a discriminant analysis is assessed by predicting the group membership of the observations and by comparing the predicted with the actual group membership. The pattern of correct and incorrect classifications can be stored in a *confusion* matrix in which the columns represent the actual groups and the row the predicted groups. At the intersection of a row and a column

is the number of observations from the column group assigned to the row group.

The performance of the model can be assessed for the observations used to compute the groups: this is the *fixed effect* model. In addition, the performance of the model can be estimated for *new* observations (i.e., observations not used to compute the model): this is the *random effect* model).

S1.17.1 Fixed Effect: Old Observations

The *fixed effect* model predicts the group assignment for the observations used to compute the barycenters of the groups. In order to assign an observation to a group, the first step is to compute the distance between this observation and all N groups. Then, the observation is assigned to the closest group. Several possible distances can be chosen, but a natural choice is the Euclidean distance computed in the factor space [6]. If we denote by $\mathbf{h}_i$ the vector of factor scores for the ith observation, and by $\mathbf{f}_n$ the vector of factor scores for the nth group, then the squared Euclidean distance (in the factor space) between the ith observation and the nth group is computed as

$$d^2\left(\mathbf{h}_i, \mathbf{f}_n\right) = \left(\mathbf{h}_i - \mathbf{f}_n\right)^{\mathsf{T}} \left(\mathbf{h}_i - \mathbf{f}_n\right) \ . \tag{17}$$

(Note that the Euclidean distance in the factor space is equivalent to the so called "chi-squared" distance in the original space). Obviously, other distances are possible (e.g., Mahalanobis distance, see for more details [6]), but the Euclidean distance has the advantage of being "directly read" on the map.

S1.17.1.1 Tolerance intervals The quality of the group assignment of the actual observations can be displayed using *tolerance* intervals. A tolerance interval encompasses a given proportion of a sample or a population. When displayed in two dimensions, these intervals have the shape of an ellipse and are called *tolerance ellipsoids*. For DICA, a group tolerance ellipsoid is plotted on the group factor score map. This ellipsoid is obtained by fitting an ellipse which includes a given percentage (e.g., 95%) of the observations. Tolerance ellipsoids are centered on their groups and the overlap of the tolerance ellipsoids of two groups reflects the proportion of misclassifications between these two groups.

S1.17.2 Random Effect: New Observations

The *random effect* model evaluates the quality of the assignment of *new* observations to groups. This estimation is obtained, in general, by using cross validation techniques that partition the data into a

learning set (used to create the model) and a *testing set* (used to evaluate the model). For DICA we use a variation of this approach called the jackknife (*a.k.a.* "leave one out") approach: Each observation is taken out from the data set, in turn, and then is projected onto the barycenter factor space computed from the remaining observations. This projection is then used to predict its group membership from the distances between the projected observation and the barycenters. In DICA the only pre-processing needed to project an observation consists into the transformation of this observation into a profile. This transformation does not require estimating parameters from the learning set, and this guarantees that the prediction of the left-out observation is random.

The assignment of an observation to a group follows the same procedure as for a fixed effect model: the observation is projected onto the group factor scores, and the observation is assigned to the closest group. Specifically, we denote $\boldsymbol{\ell}_i$ the profile vector for the ith observation, and the following matrices obtained without the ith observation are denoted (1) $\mathbf{X}_{-i}$, (2) $\mathbf{R}_{-i}$, (3) $\mathbf{B}_{-i}$ and (4) $\mathbf{W}_{-i}$ and refer to (1) the $I-1$ by J data matrix (2) the N by J barycenter matrix, (3) the N by N mass matrix, and (4) the J by J weight matrix. All these matrices are obtained using $I-1$ instead of I observations. Then the generalized eigendecomposition of $\mathbf{R}_{-i}$ is obtained as (*cf.* Equation 8):

$$\mathbf{R}_{-i} = \mathbf{P}_{-i}\boldsymbol{\Delta}_{-i}\mathbf{Q}_{-i}^{\mathsf{T}} \quad \text{with} \quad \mathbf{P}_{-i}^{\mathsf{T}}\mathbf{W}_{-i}\mathbf{P}_{-i} = \mathbf{Q}_{-i}^{\mathsf{T}}\mathbf{B}_{-i}\mathbf{Q}_{-i} = \mathbf{I} \tag{18}$$

The matrices of row and column factor scores denoted $\mathbf{F}_{-i}$ and $\mathbf{G}_{-i}$ are obtained as (*cf.* Equations 9 and 18)

$$\mathbf{F}_{-i} = \mathbf{P}_{-i}\boldsymbol{\Delta}_{-i} = \mathbf{R}_{-i}\mathbf{W}_{-i}\mathbf{Q}_{-i} \quad \text{and} \quad \mathbf{G}_{-i} = \mathbf{W}_{-i}\mathbf{Q}_{-i}\boldsymbol{\Delta}_{-i} \ . \tag{19}$$

The jackknifed projection of the ith observation, denoted $\widetilde{\mathbf{h}}_i$ is obtained (*cf.* Equation 13) as

$$\widetilde{\mathbf{h}}_i = \boldsymbol{\ell}_i\mathbf{W}_{-i}\mathbf{Q}_{-i} = \boldsymbol{\ell}_i\mathbf{G}_{-i}\boldsymbol{\Delta}_{-i}^{-1} \ . \tag{20}$$

Distances between the ith observation and the N groups can be computed (*cf.* Equation 17) with the factor scores. The observation is then assigned to the closest group. Note that the jackknife procedure assumes that there are no columns with only one non-zero entry. If there is such a column, we would create a "division by zero error" when the non-zero observation is jackknifed.

S1.17.2.1 Prediction intervals In order to display the quality of the prediction for *new* observations, we use *prediction* intervals. In order to compute these intervals, the first step is to project the jackknifed observations onto the original complete factor space. There are several ways to project a jackknifed observation onto the factor score space. Here we proposed a two-step procedure. First, the observation is projected onto the jackknifed space and is reconstructed from its projections. Then, the reconstituted observation is projected onto the full factor score solution. Specifically, a jackknifed observation is reconstituted from its factor scores as (*cf.* Equations 8 and 20):

$$\widetilde{\boldsymbol{\ell}}_i = \widetilde{\mathbf{h}}_i \mathbf{Q}_{-i}^{\mathsf{T}} \ . \tag{21}$$

The projection of the jackknifed observation is denoted $\widehat{\mathbf{h}}_i$ and is obtained by projecting $\widetilde{\boldsymbol{\ell}}_i$ as a supplementary element in the original solution. Specifically, $\widehat{\mathbf{h}}_i$ is computed as:

$$\begin{aligned} \widehat{\mathbf{h}}_i &= \widetilde{\boldsymbol{\ell}}_i \mathbf{W}\mathbf{Q} && (\textit{cf.}\ \text{Equation 9}) \\ &= \widetilde{\mathbf{h}}_i \mathbf{Q}_{-i}^{\mathsf{T}} \mathbf{W}\mathbf{Q} && (\textit{cf.}\ \text{Equation 21}) \\ &= \boldsymbol{\ell}_i \mathbf{W}_{-i} \mathbf{Q}_{-i} \mathbf{Q}_{-i}^{\mathsf{T}} \mathbf{W}\mathbf{Q} && (\textit{cf.}\ \text{Equation 20})\ . \end{aligned} \tag{22}$$

Note that $\widehat{\mathbf{h}}_i$ can also be computed from the column factor scores as

$$\widehat{\mathbf{h}}_i = \boldsymbol{\ell}_i \mathbf{G}_{-i} \boldsymbol{\Delta}_{-i}^{-2} \mathbf{G}_{-i}^{\mathsf{T}} \mathbf{W}_{-i} \mathbf{G} \boldsymbol{\Delta}^{-1} \ . \tag{23}$$

The quality of the predicted group assignment of the observations as a random model can be displayed using *prediction* intervals. A prediction interval encompasses a given proportion of the predicted elements of a sample or a population. When displayed in two dimensions, these intervals have the shape of an ellipse and are called *prediction ellipsoids.* For DICA, a group prediction ellipsoid is plotted on the group factor score map. This ellipsoid is obtained by fitting an ellipse which includes a given percentage (e.g., 95%) of the predicted observations. Prediction ellipsoids are not necessarily centered on their groups, in fact the distance between the center of the ellipse and the group represents the estimation *bias.* Overlap of two predictions intervals directly reflects the proportion of misclassifications for the "new" observations.

S1.18 Quality of the Group Separation

S1.18.1 R^2 and Permutation Test

In order to evaluate the quality of the discriminant model, we use a coefficient inspired by the coefficient of correlation. Because DICA is a barycentric technique, the total variance (i.e., the *inertia*) of the observations to the grand barycenter (i.e., the barycenter of all groups) can be decomposed into two additive quantities: (1) the inertia of the observations relative to the barycenter of their own category, and (2) the inertia of the group barycenters to the grand barycenter.

Specifically, if we denote by $\overline{\mathbf{f}}$ the vector of the coordinates of the grand barycenter (i.e., each component of this vector is the average of the corresponding components of the barycenters), the total inertia, denoted $\mathcal{I}_{\mathsf{Total}}$, is computed as the sum of the squared distances of the observations to the grand barycenter (*cf.* Equation 17):

$$\mathcal{I}_{\mathsf{Total}} = \sum_{i}^{I} m_i d^2\left(\mathbf{h}_i, \overline{\mathbf{f}}\right) = \sum_{i}^{I} m_i \left(\mathbf{h}_i - \overline{\mathbf{f}}\right)^{\mathsf{T}} \left(\mathbf{h}_i - \overline{\mathbf{f}}\right) \ . \tag{24}$$

In CA, the grand barycenter is the center of the space, and therefore $\overline{\mathbf{f}} = \mathbf{0}$ and Equation 24 reduces to

$$\mathcal{I}_{\mathsf{Total}} = \sum_{i}^{I} m_i \mathbf{h}_i^{\mathsf{T}} \mathbf{h}_i \ . \tag{25}$$

The inertia of the observations relative to the barycenter of their own category is abbreviated as the “inertia within.” It is denoted $\mathcal{I}_{\mathsf{Within}}$ and computed as

$$\mathcal{I}_{\mathsf{Within}} = \sum_{n}^{N} \sum_{i \text{ in group } n} m_i d^2\left(\mathbf{h}_i, \mathbf{f}_n\right) = \sum_{n}^{I} \sum_{i \text{ in group } n} m_i \left(\mathbf{h}_i - \mathbf{f}_n\right)^{\mathsf{T}} \left(\mathbf{h}_i - \mathbf{f}_n\right) \ . \tag{26}$$

The inertia of the barycenters to the grand barycenter is abbreviated as the “inertia between.” It is denoted $\mathcal{I}_{\mathsf{Between}}$ and computed as

$$\mathcal{I}_{\mathsf{Between}} = \sum_{i}^{I} b_n \times d^2\left(\mathbf{f}_n, \overline{\mathbf{f}}\right) = \sum_{n}^{N} b_n \times d^2\left(\mathbf{f}_n, \overline{\mathbf{f}}\right) = \sum_{n}^{N} b_n \times \left(\mathbf{f}_n - \overline{\mathbf{f}}\right)^{\mathsf{T}} \left(\mathbf{f}_n - \overline{\mathbf{f}}\right) = \sum_{n}^{N} b_n \times \mathbf{f}_n^{\mathsf{T}}\ \mathbf{f}_n \ . \tag{27}$$

So the additive decomposition of the inertia can be expressed as

$$\mathcal{I}_{\mathsf{Total}} = \mathcal{I}_{\mathsf{Within}} + \mathcal{I}_{\mathsf{Between}} \ . \tag{28}$$

This decomposition is similar to the familiar decomposition of the sum of squares in the analysis of variance. This suggest that the intensity of the discriminant model can be tested by the ratio of between inertia by the total inertia, as is done in analysis of variance and regression. This ratio is denoted R^2 and it is computed as:

$$R^2 = \frac{\mathcal{I}_{\mathsf{Between}}}{\mathcal{I}_{\mathsf{Total}}} = \frac{\mathcal{I}_{\mathsf{Between}}}{\mathcal{I}_{\mathsf{Between}} + \mathcal{I}_{\mathsf{Within}}} \ . \tag{29}$$

The R^2 ratio takes values between 0 and 1, the closer to one the better the model. The significance of R^2 can be assessed by permutation tests, and confidence intervals can be computed using cross-validation techniques such as the jackknife (see [7]) or the bootstrap (see [8]).

S1.18.2 Confidence Intervals

The stability of the position of the groups can be displayed using *confidence* intervals. A confidence interval reflects the variability of a population *parameter* or its estimate. In two dimensions, this interval becomes a confidence ellipsoid. The problem of estimating the variability of the position of the groups cannot, in general, be solved analytically and cross-validation techniques need to be used. Specifically, the variability of the position of the groups is estimated by generating *bootstraped* samples from the sample of observations. A bootstraped sample is obtained by sampling *with replacement* from the observations (recall that when sampling with replacement some observations may be absent and some other maybe repeated). The "bootstraped barycenters" obtained from these samples are then projected onto the discriminant factor space and, finally, an ellipse is plotted such that it comprises a given percentage (e.g., 95%) of these bootstraped barycenters. When the confidence intervals of two groups do not overlap, these two groups are "significantly different" at the corresponding alpha level (e.g., $\alpha = .05$). In DICA, the bootstrap can be performed directly in the factor space by sampling the elements of matrix $\mathbf{H}$ and projecting their weighted means onto the factor space.

S1.19 Where to find more information about DICA

More detailed information about DICA and related methods can be downloaded from the third author's website at www.utdallas.edu/∼herve.

References

1. Abdi H (2007) Singular Value Decomposition (SVD) and Generalized Singular Value Decomposition (GSVD). In: Salkind NJ, editor, Encyclopedia of Measurement and Statistics, Thousand Oaks, CA: Sage. pp. 907–912.

2. Abdi H, Williams LJ (2010) Principal component analysis. Wiley Interdisciplinary Reviews: Computational Statistics 2: 433–459.

3. Greenacre M (1984) Theory and Applications of Correspondence Analysis. London: Academic Press.

4. Williams LJ, Abdi H, French R, Orange JB (2010) A tutorial on multi-block discriminant correspondence analysis (MUDICA): A new method for analyzing discourse data from clinical populations. Journal of Speech Language and Hearing Research 53: 1372–1393.

5. Abdi H (2007) The eigen-decomposition: eigenvalues and eigenvectors. In: Salkind NJ, editor, Encyclopedia of Measurement and Statistics, Thousand Oaks, CA: Sage. pp. 304–308.

6. Abdi H (2007) Distance. In: Salkind NJ, editor, Encyclopedia of Measurement and Statistics, Thousand Oaks, CA: Sage. pp. 280–284.

7. Abdi H, Williams LJ (2010) Jackknife. In: Salkind NJ, editor, Encyclopedia of Research Design, Thousand Oaks, CA: Sage. pp. 655–660.

8. Efron B, Tibshirani R (1993) An Introduction to the Bootstrap. Boca Raton, FL: Chapman & Hall/CRC.

S2 Figure Captions

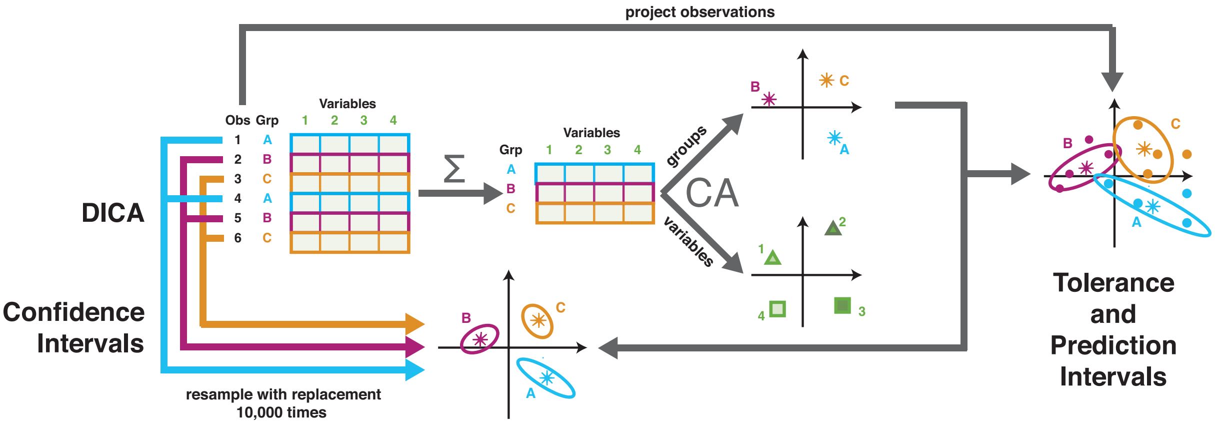

Figure S1. Schematic diagram of discriminant correspondence analysis (DICA)
